# A case report of double etiology of ecthyma gangrenosum: Pseudomonas aeruginosa and Enterococcus faecalis in an immunocompromised child occurred during influenza evolution

**DOI:** 10.1097/MD.0000000000015651

**Published:** 2019-05-17

**Authors:** Victoria Birlutiu, Rares Mircea Birlutiu, Marius Baicu, Gabriela Mariana Iancu

**Affiliations:** a Lucian Blaga University of Sibiu, Faculty of Medicine Sibiu, Academic Emergency Hospital Sibiu, Infectious Diseases Clinic; b Lucian Blaga University of Sibiu, Faculty of Medicine Sibiu; Spitalul Clinic de Ortopedie-Traumatologie si TBC osteoarticular “Foisor” Bucuresti; c Pediatric Clinical Hospital Sibiu, Intensive Care Department; d Lucian Blaga University of Sibiu, Faculty of Medicine Sibiu; Academic Emergency Hospital Sibiu - Dermatology Clinic, Sibiu, Romania.

**Keywords:** case report, double etiology, ecthyma gangrenosum, *Enterococcus faecalis*, *Pseudomonas aeruginosa*

## Abstract

**Rationale:**

Ecthyma gangrenosum is a cutaneous infection, most commonly occurring during sepsis evolution with *Pseudomonas aeruginosa* on an immunocompromised background. There have been rare case reports in previously healthy children and rarer are the cases with double etiology.

**Patient concerns:**

We present the case of a female Caucasian patient, aged 1 year and 8 months, who developed severe sepsis during flu evolution with predominant respiratory and cerebral manifestations. On admission, at skin level, there was noticed a necrotic coccygeal ulceration (with rapid increasing dimensions 0.5/0.5 cm in 24 hours), and with the transformation from a dry necrosis in a sphacelus at the periphery and progression of necrosis in depth.

**Diagnoses:**

The patient was diagnosed with ecthyma gangrenosum from which *Pseudomonsa aeruginosa and Enterococcus faecalis* were isolated from the samples that were harvested intraoperatively, decision that was taken considering the appearance of CT scan and the extremely rapid expansion of necrosis. Excisional debridement with necrectomy, lavage, and dressing being performed. The invasion of the fascia was excluded intraoperatively.

**Interventions:**

Treatment with Meropenem for 14 days was initiated, as well as amikacin and linezolid, the latter being replaced with teicoplanin for 14 days. Red blood cells transfusion, intravenous immunoglobulins, anticonvulsants were also administered.

**Outcomes:**

Under treatment the evolution was favorable.

**Lessons:**

This case brings into discussion a double etiology of ecthyma gangrenosum, in a previously healthy child that occurred in the evolution of influenza. The evolution was favorable under broad-spectrum antibiotic treatment and surgical excision.

## 1 Introduction

Ecthyma gangrenosum (EG) is a cutaneous infection most commonly associated with sepsis with *Pseudomonas aeruginosa* due to the subcutaneous thrombotic vascular lesions. In the etiology of EG, other types of bacteria may also be involved: Gram-positive and Gram-negativ cocci,^[1–4]^ Gram-negative bacteria,^[2,5–12]^ and 3 were also described cases of EG caused by fungi.^[13–15]^ The lesions of EG are caused by necrotizing vasculitis produced by the perivascular bacterial invasion in the dermis and subcutaneous tissues.

EG usually occurs in patients who are critically ill and immunocompromised. *Pseudomonas aeruginosa* may lead to sepsis in neutropenic patients or at patients with hypo- or agammaglobulinaemia, in those with aplastic anemia, malignancies, acquired immune deficiency syndrome, transplant patients, and completely unusual in previously healthy patients, and also can produce neutropenia through bone marrow suppression and inhibition of granulocyte migration.^[16–23]^

We present the case of a female Caucasian patient, who developed severe sepsis during flu evolution with predominant respiratory and cerebral manifestations. Patient that was diagnosed with ecthyma gangrenosum caused by *Pseudomonsa aeruginosa* and *Enterococcus faecalis*.

## 2 Case report

We present the case of a female Caucasian patient, aged 1 year and 8 months, without previous pathological conditions that was brought to the emergency room 1 week after onset of fever and cough, with accentuated respiratory manifestations, drowsiness, oliguria, and an intergluteal cleft lesion. At home, the patient received a treatment with amoxicillin-clavulanate, fever-reducing drugs, and oseltamivir for 2 days (in the context of a positive rapid influenza B diagnostic test).

At the time of admission, on physical examination, the following changes were noticed: the patient was febrile 38.2°C, pale, an ulceration covered by dry necrosis with hemorrhagic intergluteal border at coccyx level with a diameter of a 1.5/2 cm, accompanied by discreet local edema, bilateral bullous rales, respiration rate of 52/minute, oxygen saturation 94% to 96%, heart rate of 120 beats per minute, dry lips, palpable liver 1 cm under the coastal ribbon, and oligoanuria. The second day she presented afebrile tonic-clonic seizure, followed by choreic movements, drowsiness, neck pains, osteotendinous hyporeflexia, and plantar response in flexion. At skin level, there was noticed a rapid increase in the necrotic coccygeal ulceration (by 0.5/0.5 cm in 24 hours), with the transformation of the dry necrosis in sphacelus at the periphery and progression of necrosis in depth. On the same day, seizures repeated lasting about an hour, with spasticity of the lower limbs, eyeballs deviating upwards, and a Glasgow Coma Scale score of 7.

A lumbar puncture was performed and revealed no cytological or biochemical changes. Also an electroencephalography was performed and revealed a polymorphic theta-delta wave activity. A cranial magnetic resonance imaging (MRI) was also performed and revealed not pathological changes.

A chest radiography was performed and revealed enhancement of diffuse pulmonary interstitium, with a right infrahilar focal condensation. An thoraco-abdominal computerized tomograpohy (CT) scan was performed and revealed the following aspects: right and left posterior basal lung condensations, retroperitoneal lymph nodes with a short axis of max. 6.5 mm, agglutinated pelvic intestinal loops, rectum with thickened wall and minimal adjacent fat mass, small solution of skin continuity in the intergluteal fold in the neighborhood of the coccyx and minimal thickening of the subcutaneous tissue at this level. Bilateral inguinal adenopathy with a maximum diameter of 12/8 mm on left side were also noticed.

**Figure 1:**
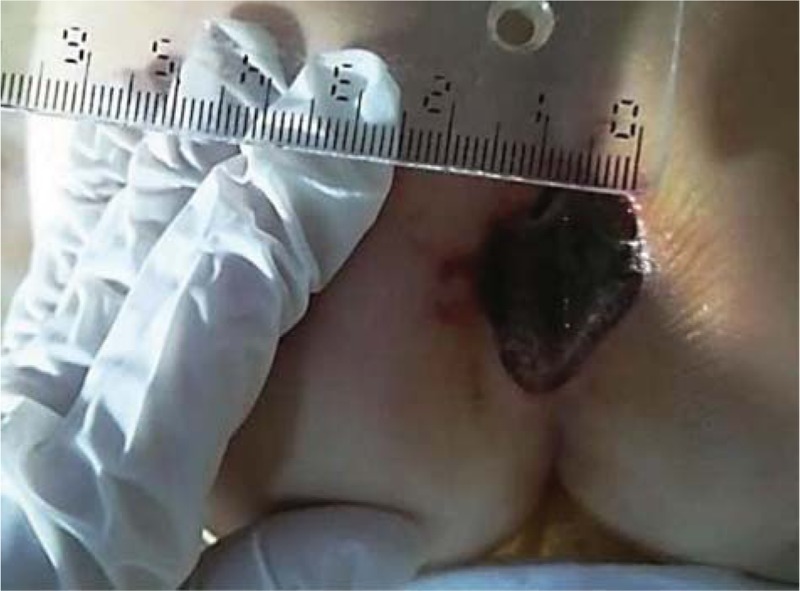
Ulceration at the time of admission.

**Figure 2:**
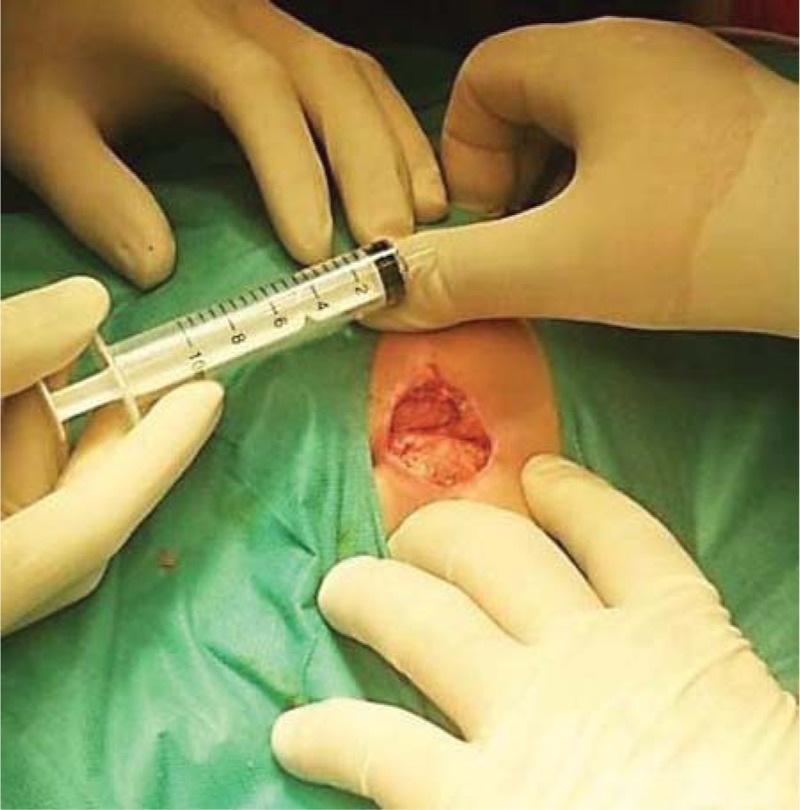
Surgical debridement of necrosis without affecting the fascia.

**Figure 3:**
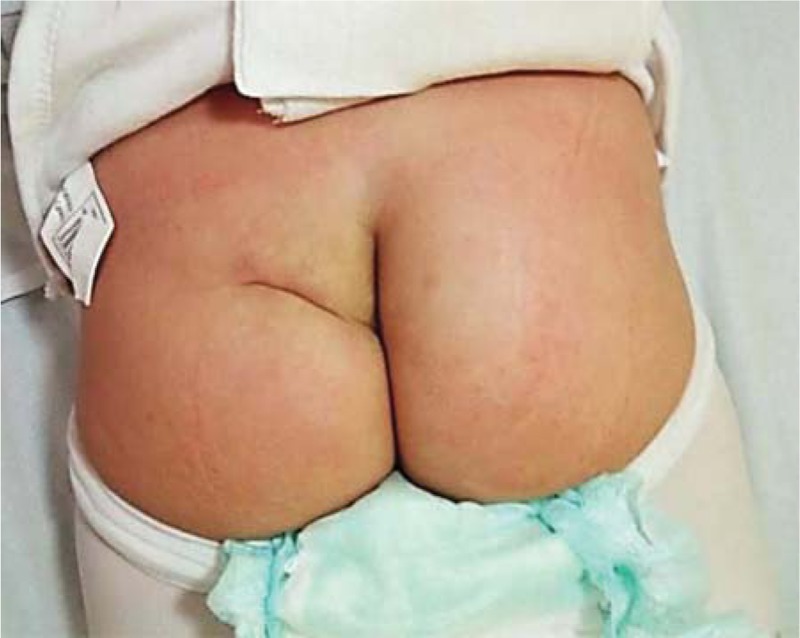
Clinical appearance at 2 months after admission, after surgical restoration of the skin defect.

Considering the appearance of CT scan and the extremely rapid expansion of necrosis, it is decided that the patient to undergo surgical intervention of excisional debridement with necrectomy, lavage, and dressing. Intraoperatively, invasion of the fascia was excluded and tissue samples were harvested for microbacteriological examination (Figs. 1–3).

Bacteriological examination of the tissues sampled intraoperatively was positive for *Pseudomonas aeruginosa* and *Enterococcus faecalis* confirming the diagnosis of diagnosis of ecthyma gangrenosum, strains that were identified using the VITEK 2 Compact analyzer (bioMérieux, Marcy-l’Étoile, France). The MICs (minimum inhibitory concentrations) were assessed according to the European Committee on Antimicrobial Susceptibility Testing breakpoints. The isolated strain of *Pseudomonas aeruginosa* was sensitive to ceftazidime, cefepime, amikacin, gentamicin, tobramycin, ciprofloxacin, pefloxacin, imipenem, meropenem, ticarcillin-resistant, colistin, trimethoprim/sulfamethoxazole, and intermediate sensitive to piperacillin-tazobactam. The *Enterococcus faecalis* strain was sensitive to ciprofloxacin, moxifloxacin, ampicillin, vancomycin, teicoplanin, tigecycline, tetracycline resistant, streptomycin, clindamycin, erythromycin, and intermediate sensitive to trimethoprim/sulfamethoxazole.

Although blood cultures were negative, with increased levels of procalcitonin (4.35 ng/ml, references values: 0–0.5 ng/dl) and C-reactive protein (200 mg/dl, references values: 0–10 mg/dl), concomitant pulmonary, cerebral disease, and renal failure supported the diagnosis of sepsis. At the time of admission and during hospitalization, the patient did not experience neutropenia, neutrophil counts ranging from 7550 to 9220/mm^3^, but the patient had anemia (Hemoglobin: 8.2–6.2–9 g/dl, references values: 13–17 g/dl) and thrombocytopenia (92,000–96,000/mm^3^, references values: 150–400,000/μl), which were normalized during the evolution. Upon discharge, acute renal failure was corrected under conservative treatment (creatinine 1.57 mg/dl, references values: 0.7–1.2 mg/dl; blood urea nitrogen 104.9 mg/dl, references values: 16.6–48.5 mg/dl).

## 3 Discussions

Ecthyma gangrenosum (EG) is a skin lesion most commonly associated with sepsis caused by *Pseudomonas aeruginosa*, a consequence of subcutaneous thrombotic vascular lesions. In the etiology of EG, other types of bacteria may also be involved: Gram-positive cocci, such as *Streptococcus pyogenes*,^[1,2]^*Staphylococcus aureus*,^[3]^ and Gram-negative cocci: *Neisseria gonorrhoea*,^[4]^ or Gram-negative bacteria: *E coli*,^[5]^*Citrobacter freundii*,^[2]^*Klebsiella pneumoniae*,^[6]^*Morganella morganii*,^[7]^*Burkholderia cepacia*, *Pseudomonas stutzeri*,^[8]^*Serratia marcescens*,^[9]^*Xanthomonas maltophilia*,^[10]^*Aeromonas hydrophila,*^[11]^*Chromobacterium violaceum*.^[12]^ There are also described cases of EG caused by fungi: *Candida albicans*,^[13]^*Aspergillus fumigatus, Fusarium solani,*^[14]^ and *Mucor pusilus*.^[15]^

*Pseudomonas aeruginosa* may lead to sepsis in neutropenic patients,^[16]^ in patients with hypo- or agammaglobulinaemia,^[17]^ in those with aplastic anemia, malignant haemopathies, acquired immune deficiency syndrome (HIV/AIDS), hepatic, pancreatic, and small intestine transplant^[18]^ and completely unusual in previously healthy patients. In the latter, there are described cases of EG associated with *Pseudomonas aeruginosa* predominantly in males and infants,^[19]^ in patients with viral infections, with transient neutropenia, and due to the recent use of ineffective antibiotics against *Pseudomonas aeruginosa*. The primary site *of Pseudomonas aeruginosa* may be in the airways, the digestive tract or the urinary tract, in the context of bacteremia.^[18,20–22]^*Pseudomonas aeruginosa* toxins can produce neutropenia through bone marrow suppression and inhibition of granulocyte migration.^[22,23]^ Severe infections can be associated with invasive procedures, ventilation pneumonia, cystic fibrosis, extensive burns etc.

The absence of neutropenia and a condition responsible for immunodepression make us believe that in the case presented, *Pseudomonas aeruginosa* was responsible for transient neutropenia. The respiratory manifestations that dominated the clinical picture suggest that the primary site of the infection was the respiratory tract.

The initial phase of the inflammatory immune response that characterizes the viral sepsis or a severe influenza infection commonly is followed by a period of immune suppression. Immune suppression that if it is associated with prolonged and excessive inflammatory response results in poor outcomes. The immune suppression is characterized by a decreased function in both innate and adaptive immunity, being associated with an increased expression of negative co-stimulatory molecules and decreased expression of positive co-stimulatory molecules. It is also associated with a decrease in T cell exhaustion and cell apoptosis and with an increased regulatory T cell expression and numbers of myeloid-derived suppressor cells. The result being a directly lead to severe sepsis or an in increased risk of infections from secondary pathogens, reactivation of dormant infections, and/or natural microbiota becoming pathogenic.^[24,25]^

The particularity of the case consisted in the neurological manifestations, with choreic movements, which disappeared under the antibiotic and anticonvulsant medication. The initiation of broad-spectrum treatment has been associated with the favorable evolution of the case, especially since it is known that ineffective treatment can be the unfavorable determining factor.^[26–30]^

Although the association of a beta-lactam and an aminoglycoside for the treatment of these cases is considered to be optimal, the use of an anti-pseudomonas cephalosporin may be associated with previous resistance to cephalosporins,^[31]^ aminoglycosides may not be acceptable due to renal insufficiency present, or it may be an association of *Pseudomonsa aeruginosa* with other bacteria, the combination of which is ineffective (in this case with *Enterococcus*). Even the use of pseudomonas beta-lactam penicillin as a therapeutic option may be associated with the risk of resistance (as in this case).^[32]^

Treatment with Meropenem for 14 days was initiated, as well as amikacin and linezolid, the latter being replaced with teicoplanin for 14 days. Red blood cells transfusion, intravenous immunoglobulins, anticonvulsants were administered in parallel with surgical local therapy with favorable evolution. EG cases with sepsis are accompanied by an important death rate of 38% to 77%, respectively of 15%, for the non-septic patient.

A single case has been described with the association of *Pseudomonas aeruginosa* and vancomycin-resistant *Enterococcus faecalis* EG in a 23-month-old child with immunosuppressive therapy and triple transplantation,^[18]^ not in a previously healthy child.

## 4 Informed consent

Written informed consent was obtained from the patient‘s parents for publication of this case report and any accompanying images. The study was accepted by the Ethics Committee of the hospital and they encouraged publishing the article. A copy of the written consent is available for review by the Editor-in-Chief of this journal.

## 5 Conclusions

This case brings into discussion a double etiology of EG, caused by *Pseudomonas aeruginosa* resistant to anti-pseudomonal beta-lactam penicillin and *Enterococcus faecalis*, in a previously healthy child under recent treatment with amoxicillin-clavulanate and oseltamivir, occurred in the evolution of influenza. Although neutropenia was not identified, being possibly corrected after the introduction of antiviral medication, the case suggests the need to initiate wide-spectrum antibiotics until obtaining the antibiogram and after de-escalation.

## Author contributions

All authors contributed equally to this manuscript in terms of acquisition, analysis and interpretation of data, conception and design, drafting the manuscript. All authors read and approved the final manuscript.

**Conceptualization:** Victoria Birlutiu, Rares Mircea Birlutiu, Gabriela Mariana Iancu.

**Formal analysis:** Victoria Birlutiu, Rares Mircea Birlutiu, Marius Baicu, Gabriela Mariana Iancu.

**Investigation:** Victoria Birlutiu, Rares Mircea Birlutiu, Marius Baicu.

**Methodology:** Victoria Birlutiu, Rares Mircea Birlutiu, Marius Baicu, Gabriela Mariana Iancu.

**Resources:** Victoria Birlutiu, Rares Mircea Birlutiu, Gabriela Mariana Iancu.

**Supervision:** Victoria Birlutiu, Rares Mircea Birlutiu.

**Validation:** Victoria Birlutiu, Rares Mircea Birlutiu, Gabriela Mariana Iancu.

**Visualization:** Victoria Birlutiu, Rares Mircea Birlutiu, Marius Baicu, Gabriela Mariana Iancu.

**Writing – original draft:** Victoria Birlutiu, Rares Mircea Birlutiu.

**Writing – review & editing:** Victoria Birlutiu, Rares Mircea Birlutiu.
